# Supplementary material for: The invasion pore induced by Toxoplasma gondii
Source: bioRxiv. 2024 Oct 13:2024.10.11.617945. Preprint. [Version 1] doi: 10.1101/2024.10.11.617945 (PMC11482919; doi:10.1101/2024.10.11.617945)
Supplement: Supplementary file 1 [file media-1.pdf]

The invasion pore induced by *Toxoplasma gondii*

Kegawa Y.,[1*] Male F.[2*], Jiménez-Munguía I.,[1] Blank P.S.,[1] Mekhedov E.,[1] Ward G.,[2**] Zimmerberg J.[1**]

[1] Section on Integrative Biophysics; Division of Basic and Translational Biophysics, *Eunice Kennedy Shriver* National Institute of Child Health and Human Development (NICHD), National Institutes of Health (NIH)

[2] Department of Microbiology and Molecular Genetics, University of Vermont Larner College of Medicine, Burlington, United States.

[*]Contributed equally [**]Corresponding Authors

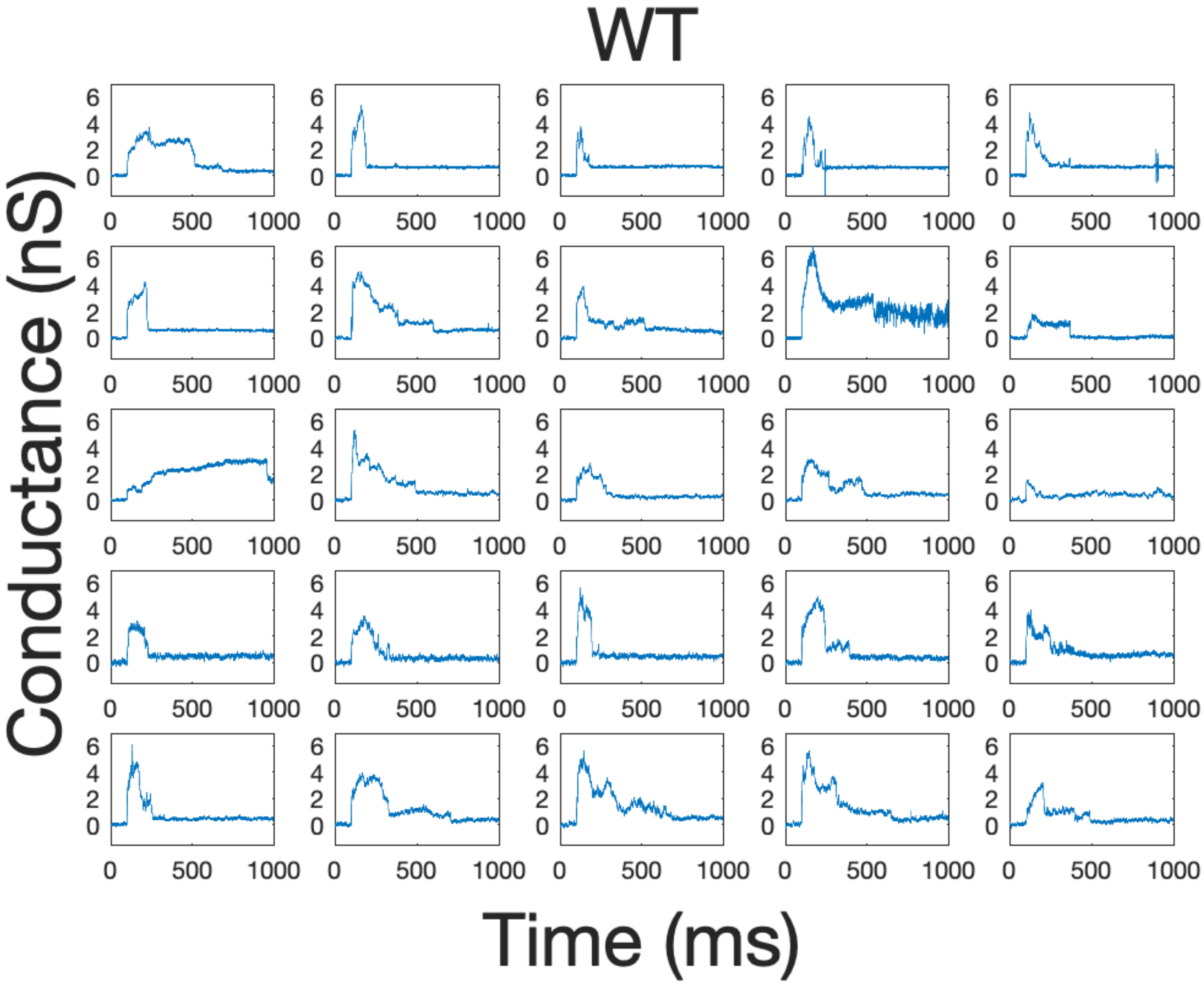

Supplementary Figure 1. Gallery of 25 WT parasite conductance transients calculated from recorded current measured using -60 mV holding potential.

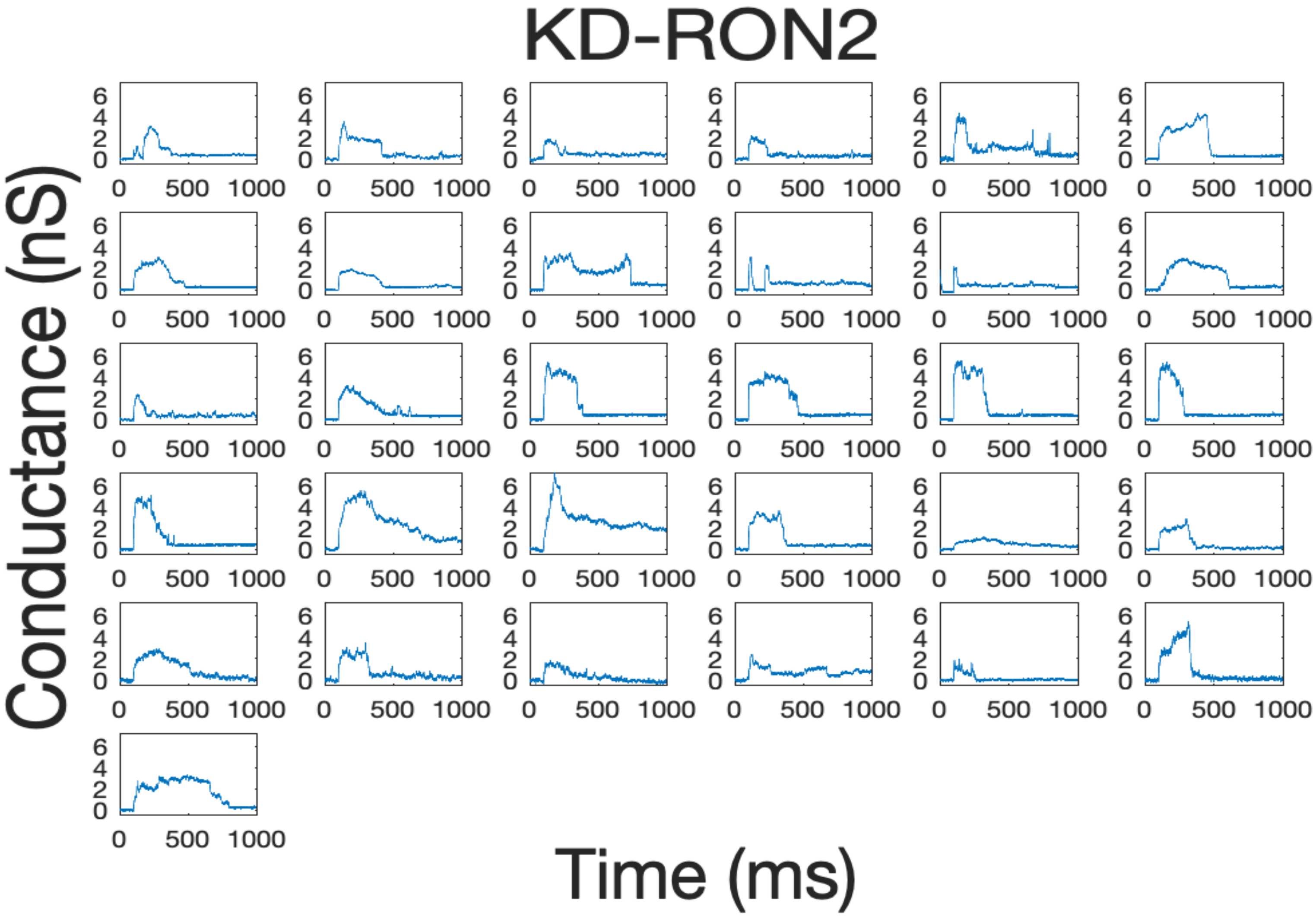

Supplementary Figure 2. Gallery of 31 KD-RON2 parasite conductance transients calculated from recorded current measured using -60 mV holding potential.

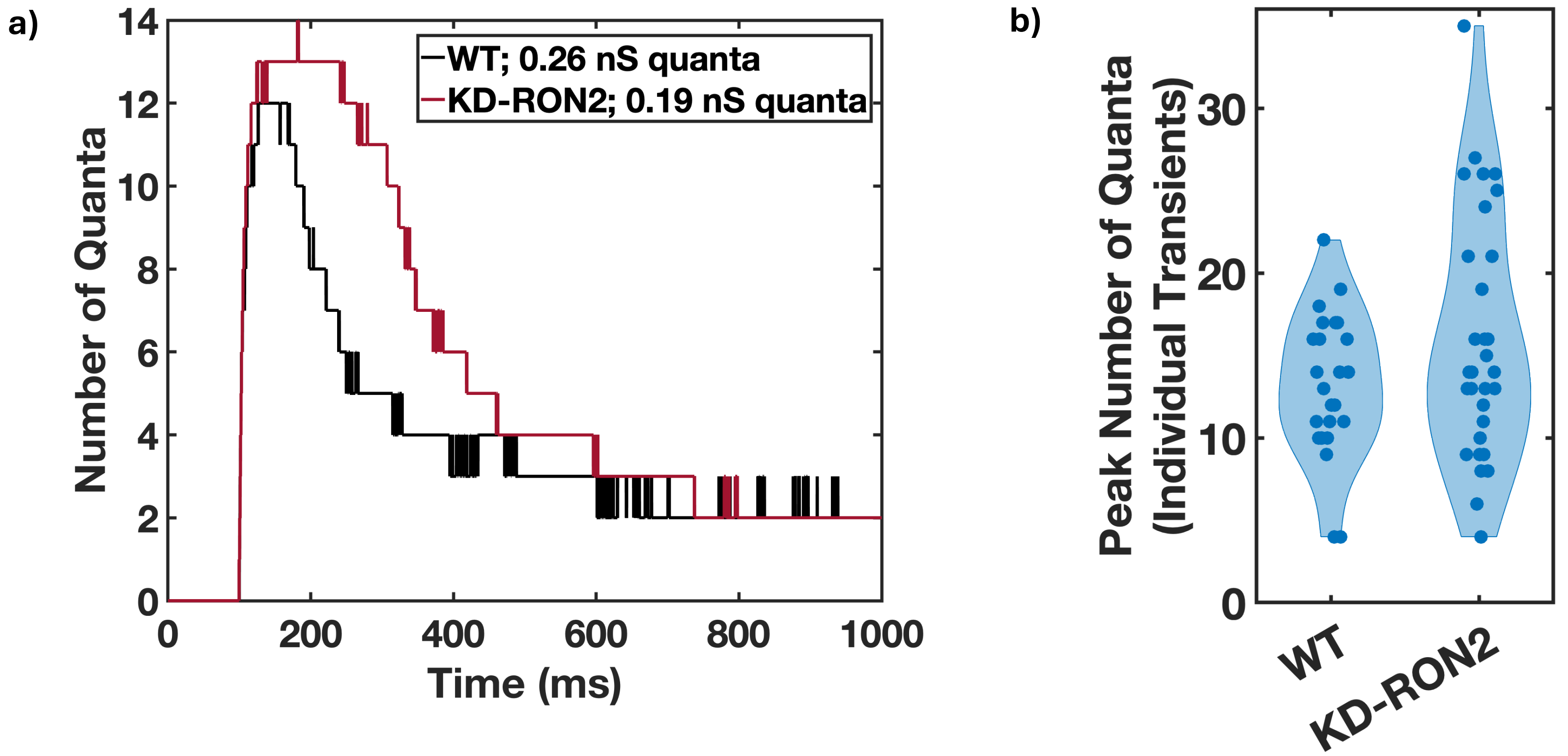

Supplementary Figure 3. The same number of quantal units contribute to the conductance maxima of WT and KD-RON2 transients. a) Transformation of the mean transient conductance (Figure 3a) using the peak quantal sizes for WT (0.26 nS) and KD-RON2 (0.19 nS), rounded to nearest integer. b) Violin plots of the number of quantal units at the maximum conductance of WT and KD-RON2 transients (n = 25 and 31, respectively).

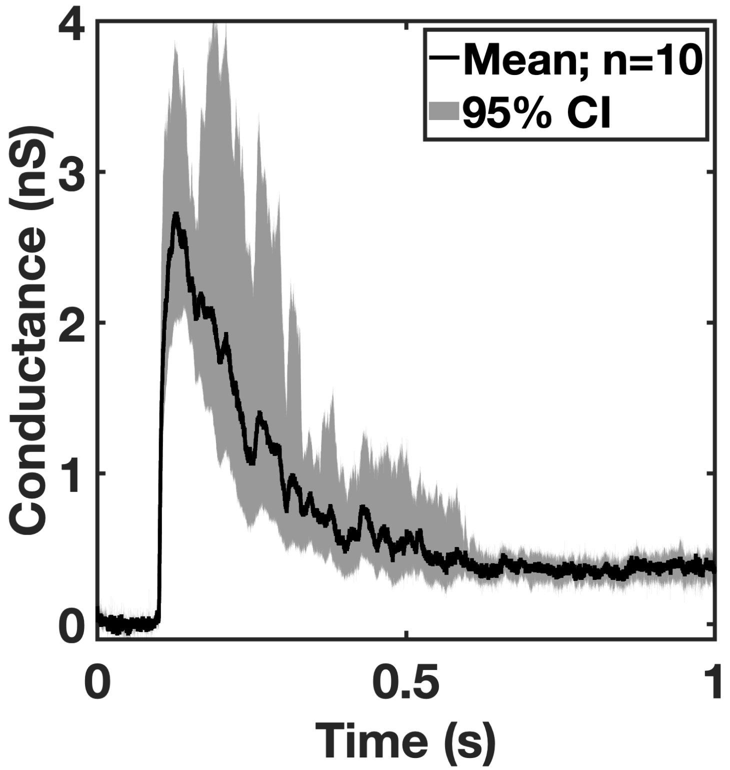

Supplementary Figure 4. Transients induced by WT parasites in low external calcium concentration (0.1 mM $CaCl_2$) have similar properties to WT transients induced in regular LCIS (2.0 mM $CaCl_2$; Figure 3a). The average waveform of low-calcium transients displays the characteristic fast rise to peak conductance and slower recovery to a new baseline (n=10).

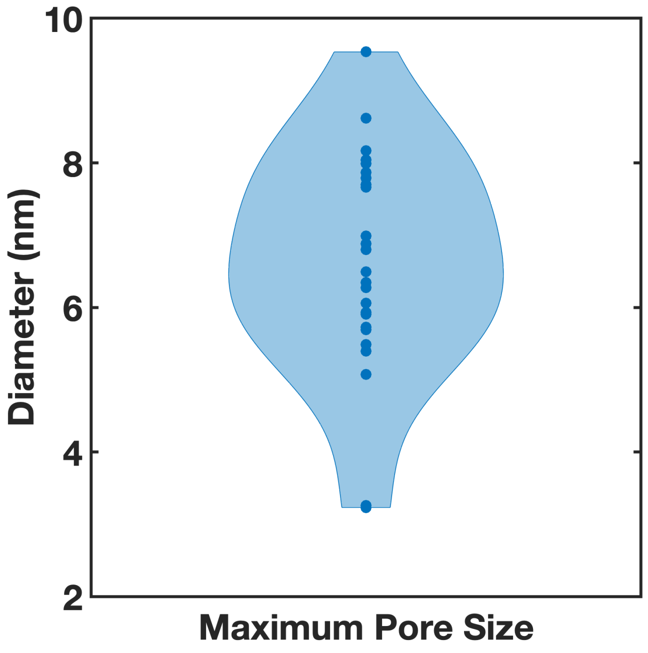

Supplementary Figure 5. Violin plot of pore diameters calculated from individual WT transient maximum conductance using a model for a cylindrical pore (see methods).
